# Tungsten Accumulation in Hot Spring Sediments Resulting from Preferred Sorption of Aqueous Polytungstates to Goethite

**DOI:** 10.3390/ijerph182312629

**Published:** 2021-11-30

**Authors:** Qian Zhao, Qinghai Guo, Li Luo, Ketao Yan

**Affiliations:** State Key Laboratory of Biogeology and Environmental Geology and School of Environmental Studies, China University of Geosciences, Wuhan 430074, China; qianz0607@gmail.com (Q.Z.); rolyvip@gmail.com (L.L.); yanktwork@gmail.com (K.Y.)

**Keywords:** polytungstate, hot spring, goethite, adsorption, environmental geochemistry, Rehai

## Abstract

Geothermal waters usually have elevated tungsten concentrations, making geothermal systems important sources of tungsten in the environment. To study the transport of tungsten in hot springs to hot spring sediment, which is one of the key processes for the release of geothermally derived tungsten to the surface environment, geochemical investigations of the hot springs and their corresponding sediments in Rehai (a representative hydrothermal area in southwestern China) and systematic laboratory experiments of tungstate and polytungstate adsorption onto typical iron-bearing minerals in hot spring sediments (i.e., pyrite and goethite) were conducted. The results demonstrate that considerable tungsten concentrations (i.e., not much less than 10 µg/L), formation of polytungstates under acidic conditions, and enrichment of iron oxide minerals represented by goethite are the prerequisites for extreme enrichment of tungsten in hot spring sediments (e.g., 991 µg/g in the ZZQ spring outflow channel). The absence of any of these conditions would weaken the immobilization of aqueous tungsten and result in higher mobility of tungsten in the hot springs and its further transport downstream, possibly polluting the other natural waters in and around Rehai that serve as local drinking water sources. This study provides an insight for identifying the key geochemical processes controlling the transport and fate of undesirable elements (in this case, tungsten) in geothermal systems.

## 1. Introduction

In recent years, environmental toxicology studies of tungsten (W) have overturned the misconception that tungsten is non-toxic [1,2,3,4]. Although the pathological relationship between the enrichment of tungsten in environmental media and human diseases (e.g., leukemia in children living near a tungsten mining area in Nevada, USA) remains unclear [5,6,7], a clinical study has shown that a high concentration of tungsten in human urine can be an indicator of stroke or cardiovascular disease [8]. Animal experiments have also confirmed the toxicity and carcinogenicity of tungsten [9,10,11,12,13]. In addition, tungsten has relatively high mobility in aqueous environments [1,14,15]. Therefore, geochemical research on tungsten from an environmental toxicology perspective is of critical importance. 

Sources of tungsten can generally be divided into two categories: natural processes and anthropogenic activities. The former category includes the release of tungsten from magmatic fluids [16,17], weathering of tungsten-rich minerals [18], desorption of tungsten from iron or manganese oxides/hydroxides [19,20], etc. The latter category includes studded tires and deicing salts used in snowy conditions [21], ammunition [14], agricultural fertilizer application, domestic sewage discharge [22,23], etc. In contrast to other natural waters (e.g., meteoric, seawater, and river), geothermal waters usually have higher tungsten concentrations due to the input of magmatic fluid and the lithology of reservoir host rocks [24], making geothermal systems one of the important sources of tungsten in the environment. The tungsten contents of submarine hydrothermal fluids can exceed the background values for seawater (<0.2–1 ng/L) by 1–4 orders of magnitude [25,26]. For example, in the submarine hydrothermal fluids of the Central Indian Ridge, North Pacific Ocean, and East China Sea, the tungsten concentrations can be up to 0.039, 2.8, and 22.6 µg/L, respectively [27]. A higher tungsten concentration of 224.5 µg/L was found in the hot springs in the South Nahanni area of Canada [28]. Concentrations of tungsten can be as high as 300 µg/L in the geothermal waters near tungsten deposit areas [3]. Elevated tungsten concentrations in non-geothermal surface and ground waters as a result of the confluence of tungsten-rich geothermal waters are also commonly observed [28,29]. However, compared to the studies conducted on other sources of tungsten in the environment (e.g., release from mining activities), there has been much less research on tungsten from geothermal systems. 

In terms of the environmental geochemical studies of tungsten, magmatic geothermal water is of greater significance than other types of geothermal waters due to its unique chemical characteristics (e.g., wide pH range and high sulfide contents), possibly resulting in the occurrence of various tungsten species (e.g., polytungstates and thiotungstates) other than monotungstate [24,30,31]. Tungsten speciation in water is an important factor influencing its environmental transport and fate [15,32]. Polymeric tungstates were reported to be more mobile than monomeric tungstate, and their formation at low pH (4–7) significantly inhibited the adsorption of tungsten by ferrihydrite and facilitated the migration of tungsten in the aqueous environment [33]. Indoor column leaching experiments also indicated that the formation of polymeric tungstates can substantially reduce the soil/water partition coefficient of tungsten [34]. Therefore, in-depth study of the influence of tungsten speciation in magmatic geothermal water on its environmental behavior is of great significance. 

The Rehai geothermal area, located in the Tengchong volcanic region, is a representative magmatic geothermal area in mainland China with a wide pH range (1.85–9.96) and relatively high tungsten concentrations (up to 87.3 µg/L) in hot spring waters. In our previous research, the migration patterns of tungsten in the Rehai hot spring waters to the hot spring sediments were preliminarily summarized [24]. However, the influences of the tungsten speciation in the hot springs and the mineralogy of the hot spring sediments on the geochemical transport of geothermal tungsten have not been studied in depth. Therefore, the objectives of this study were to explore the interaction mechanisms between the main iron-bearing minerals (goethite (FeOOH) and pyrite (FeS_2_)) in the hot spring sediments and the different tungsten species (tungstate and polytungstates) in the hot spring waters to identify the predominant factors controlling the geochemical transport and fate of geothermal tungsten and to elucidate the importance of environmental studies of geothermal tungsten for preventing tungsten pollution related to geothermal water discharge.

## 2. Materials and Methods

### 2.1. Study Area, Geochemical Sampling, and Analyses of Collected Samples

The Tengchong geothermal region is located in the Yunnan–Sichuan–Tibet Geothermal Province; this is the sole high-temperature geothermal belt in mainland China [24]. Rehai is a geothermal area in Tengchong characterized by the most active and diversified hydrothermal system. It has a magma chamber at a depth of around 7 km as its heat source [35]. Leaching of reservoir rocks (basically granites with tungsten contents as high as 8.4 ppm) at high temperatures and input of magmatic fluids released from the magma chamber are responsible for the generally high tungsten concentrations of the geothermal waters at Rehai [24]. Tungsten is more enriched in granite than in other common rocks such as basalt (0.21–0.9 ppm), diabase (0.31 ppm), andesite (0.47–0.56 ppm), rhyolite (1.5 ppm), shale (0.79–1.4 ppm), sandstone (1.16 ppm), limestone (0.67 ppm), and dunite (0.024 ppm) [36,37]. Strong surface hydrothermal manifestations are ubiquitous in Rehai, including hydrothermal explosions, boiling springs, hot springs, fumaroles, and steaming ground. Almost all of the hot springs discharge into the Zaotang River running through Rehai.

**Figure 1:**
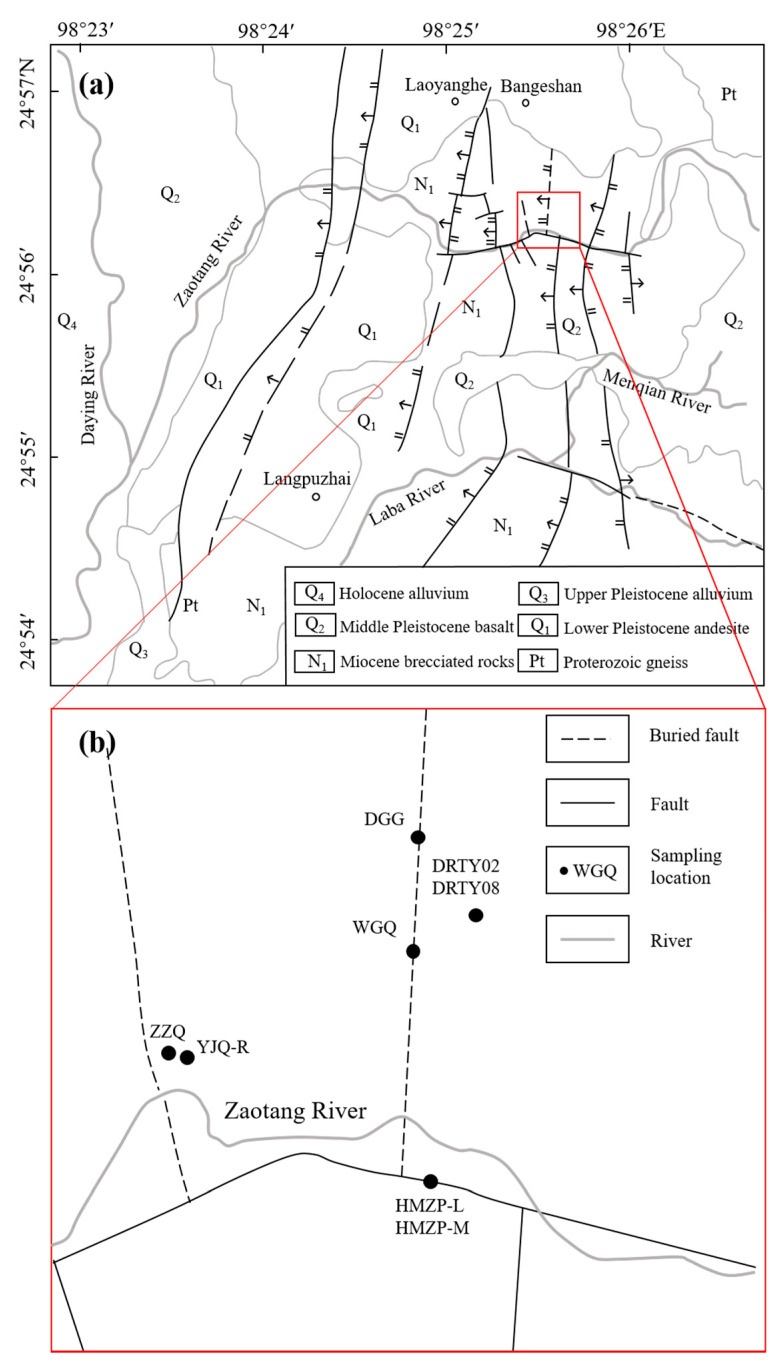
Simplified geological map of the Rehai geothermal field based on [40] (**a**) and sampling locations (**b**).

In this study, a total of 16 water samples were collected from the Rehai hot spring vents and their outflow channels (Figure 1). Unstable chemical parameters, including temperature, pH, and electrical conductivity (EC), were measured in situ with a portable water quality analyzer (Hach LDOTM HQ10, Loveland, CO, USA). Sulfide concentration and alkalinity were measured on-site using the methylene blue method (Hach Sension 2, Loveland, CO, USA) and the Gran titration method [38], respectively. The HCO_3_^−^ and CO_3_^2−^ concentrations were calculated from the measured values of alkalinity and pH using PHREEQC incorporating the WATEQ4F database [39]. Water samples filtered through 0.45-micrometer membranes for laboratory analyses were collected in high-density polyethylene bottles washed three times with the water samples beforehand. For the analysis of total metal concentrations, reagent-grade HNO_3_ was added to one sample split collected at each site to decrease the pH below 1, while no chemical agents were added to sample splits for the analysis of major anions. Note that to avoid the potential precipitation of tungsten oxide under acidic conditions, the analysis of W was conducted using the sample splits without any acidification. All the samples were stored at 4 °C and analyzed within 2 weeks after sampling. The concentrations of F, Cl, and SO_4_ were measured by ion chromatography (ThermoFisher Scientific; ICS 900, Waltham, MA, USA); those of K, Ca, Na, and Mg using an inductively coupled plasma optical emission spectrometer (ThermoFisher Scientific; IRIS intrepid II XSP, Waltham, MA, USA); and those of Fe and W using an inductively coupled plasma mass spectrometer (ThermoFisher Scientific; iCAP RQ). The limits of detection (LODs) and the limits of quantification (LOQs) for elemental analyses are estimated in Table S1. PHREEQC modeling was performed using the WATEQ4F database updated with the chemical thermodynamic parameters of various tungsten species (Table S2) to calculate the tungsten speciation in the water samples based on their chemical compositions.

In addition, sediment samples were collected at the sites where the geothermal waters were sampled and stored in sealed plastic bags at −20 °C. The contents of Fe and W in these sediment samples were determined via total digestion (HF-HNO_3_) followed by inductively coupled plasma mass spectrometry (ThermoFisher Scientific; iCAP RQ) analysis [41]; analyses of rock standards are listed in Table S3.

### 2.2. Formation of Polytungstates and Their Identification by UV-Vis

The formation and stability of polytungstates were determined by detecting the changes in tungsten polymerization in solution using UV spectroscopy on the basis of the fact that (1) the UV absorption spectra of hexameric, decameric, and dodecameric tungstates in solution are significantly different [42,43], and (2) the relationship between solution absorbance and tungsten concentration at specific wavelengths is in accordance with the Beer–Lambert law. Three groups of solutions with initial tungsten concentrations of 1, 10, and 100 µmol/L were prepared with Na_2_WO_4_·2H_2_O at room temperature. One group was not subject to pH adjustment, and the other two groups were adjusted to pH 3 and 9 with HCl and NaOH, respectively. Water samples were taken at 1, 6, 12, and 24 h from the prepared solutions. The absorbance of each sample was tested using an ultraviolet–visible (UV-Vis) spectrophotometer (HITACHI U-3900), with the thickness of the quartz cuvette equal to 1 cm, ultrapure water as the blank, and the wavelength range 220–380 nm.

### 2.3. Tungsten Sorption Experiments

#### 2.3.1. Reagents and Materials

All the chemicals used for the experiments were of analytical purity and from Sinopharm Chemical Reagent Co., Ltd (Shanghai, China), except for 3Na_2_WO_4_·9WO_3_·H_2_O (Alfa Aesar, CAS: 12141-67-2, Waltham, MA, USA). All experiments were performed in a glove box under a nitrogen atmosphere to maintain anoxic conditions. Experimental solutions were prepared with ultra-pure water that was deoxygenated by bubbling high-purity nitrogen gas (99.999% purity) through a diffusion tube for at least 1 h within a glove box filled with a N_2_ atmosphere. Monotungstate and polytungstate solutions were prepared by dissolving sodium tungstate dihydrate (i.e., Na_2_WO_4_·2H_2_O) and sodium polytungstate (i.e., 3Na_2_WO_4_·9WO_3_·H_2_O), respectively, in deoxygenated water. Sodium chloride (i.e., NaCl) was used to adjust the ionic strength values of the solutions. The preparation of goethite and the pretreatment of pyrite are described in the (Supplementary Figure S1).

#### 2.3.2. Kinetic Experiments

The batch experiments to investigate the adsorption kinetics of monotungstate and polytungstate onto goethite and pyrite were carried out by reacting 100 mL of feed solutions with the same tungsten concentration of 100 μmol/L and 20 mmol/L NaCl background electrolyte in polyethylene bottles with 0.178 g goethite or 0.176 g pyrite at 25 °C. The supernatants of water samples taken at reaction times of 0.25, 0.5, 1, 2, 4, 6, 8, 12, 18, and 24 h were filtered through a 0.22 µm pore size filter and stored at 4 °C until the measurement of tungsten concentration within one week.

The adsorption kinetics of tungsten onto goethite and pyrite were analyzed using two widely used models, i.e., a pseudo-first-order model [44] (Equation (1)) and a pseudo-second-order model [45] (Equation (2)).

(1) $$\log\left(q_{e}-q_{t}\right)=\log q_{e}-\frac{k_{1}}{2.303}\cdot t$$

(2) $$\frac{t}{q_{t}}=\frac{1}{k_{2}\cdot q_{e}^{2}}+\frac{1}{q_{e}}\cdot t$$

where *q_e_* and *q_t_* (mg/g) are the amounts of tungsten adsorbed onto iron-bearing minerals at equilibrium and at time *t* (h), respectively, and *k_1_* and *k_2_* (h^−1^) are the adsorption rate constants.

#### 2.3.3. Adsorption Isotherms

The kinetic experiments indicated that an equilibration time of 24 h was sufficient for attaining equilibrium. Thus, 100-milliliter suspensions were reacted for 24 h at 25 °C with 0.178 g goethite or 0.176 g pyrite and 20 mmol/L NaCl background electrolyte containing monotungstate or polytungstate with a concentration range of 0.1–100 μmol/L. After a 24-h reaction period, the supernatants were sampled, filtered through a 0.22 µm pore size filter, and stored at 4 °C until measurement of the tungsten concentration within one week.

The Freundlich [46] (Equation (3)) and Langmuir [47] (Equation (4)) isotherm models were applied to fit the experimental data. The Langmuir model is commonly used for delineating ideal and monolayer adsorption, while the Freundlich model is valid for non-ideal and multilayer adsorption.

(3) $$q_{e}=K_{F}\cdot c_{e}^{1/n}$$

(4) $$\frac{c_{e}}{q_{e}}=\frac{1}{K_{L}\cdot q_{m}}+\frac{c_{e}}{q_{m}}$$

where *q_e_* is the amount of tungsten adsorbed onto iron-bearing minerals at equilibrium (mg/g), *c_e_* is the equilibrium concentration of tungsten in solution (mg/L), *K_F_* and *n* are the Freundlich constants, *K_L_* is the Langmuir constant (L/mg) related to binding energy, and *q_m_* is the saturated adsorption capacity (mg/L).

#### 2.3.4. Effects of Temperature and Ionic Strength

To study the effects of temperature on the adsorption, batch experiments were carried out in 100 mL 0.02 mol/L NaCl background electrolyte solutions with 100 μmol/L monotungstate or polytungstate and 0.178 g goethite at 25, 45, 65, and 85 °C. The same batch experiments were conducted but using 0.176 g pyrite instead of goethite. To investigate the effects of ionic strength, batch experiments were conducted in 100 mL 0.02, 0.1, and 0.2 mol/L NaCl background electrolyte solutions with 100 μmol/L monotungstate or polytungstate and 0.178 g goethite or 0.176 g pyrite at 25 °C. After a 24-hour reaction period, the supernatants were sampled, filtered through a 0.22 µm pore size filter, and stored at 4 °C until measurement of the tungsten concentration within one week. The reacted solid samples were characterized by X-ray powder diffraction (XRD), scanning electron microscopy equipped with energy-dispersive X-ray spectrometry (SEM-EDX), and X-ray photoelectron spectrometry (XPS) after being dried in a vacuum drying oven.

#### 2.3.5. Measurements and Data Analysis

The compositions of the solid samples were characterized by XRD (TD3500, Dandong Tongda, Dandong, China) at 30 Kv and 20 mA with scanning angles of 5–90° as well as a step size of 0.04° and a sampling time of 0.5 s. The collected XRD patterns were analyzed by JADE 7 software (Materials Data Inc., Livermore, CA, USA). The surface morphology and elemental compositions of the solid samples were analyzed by SEM-EDX (FEI Quanta 200, FEI, Hillsboro, OH, USA). The elemental compositions and chemical bonding information of the solid samples were analyzed by XPS (ESCALAB 250 Xi, ThermoFisher Scientific, Waltham, MA, USA) (Al K_α_ = 1486.6 eV with a step of 0.05 eV). All spectra were charge-corrected using the C1s line at 284.8 eV. Avantage software (ThermoFisher Scientific, Waltham, MA, USA) was used for peak splitting and fitting processing, and the peaks were considered to belong to the same chemical bond when they varied within ±0.3 eV.

Solution pH was measured with a pH meter (FE28, Mettler Toledo, Zurich, Swiss) calibrated with standard solutions. The tungsten concentration in solution was determined using inductively coupled plasma mass spectrometry (iCAP RQ, ThermoFisher Scientific, Waltham, MA, USA). The amount of tungsten adsorbed onto the solid samples (*q_e_*) at equilibrium was determined using the mass balance equation (Equation (5)). The tungsten adsorption rate (R, %) was calculated using Equation (6).

(5) $$q_{e}=\frac{\left(c_{o}-c_{e}\right)\cdot v}{m}$$

(6) $$R=\frac{c_{o}-c_{e}}{c_{o}}\cdot 100\%$$

where *c_o_* and *c_e_* are the initial and equilibrium concentrations, respectively, of tungsten in the aqueous phase (mg/L); *v* is the volume of solution (L), and *m* is the mass of adsorbent (g). 

## 3. Results

### 3.1. General Geochemistry of the Hot Springs and Tungsten in the Hot Spring Sediments

The on-site parameters and major chemical constituents of the Rehai hot spring samples are presented in Table 1 and Table S4. The pH values of the samples varied widely between 2.78 and 9.15, and they were divided into acidic (pH < 7) and neutral/alkaline (pH ≥ 7) springs using pH = 7 as the threshold value. The piper diagram (Figure S2) shows that the hydrochemical types of the acidic hot spring samples (nine samples) were mainly SO_4_-Na and SO_4_-Cl-Na, while that of the neutral/alkaline hot spring samples (seven samples) was Cl-HCO_3_-Na.

The differences in the hydrogeochemical characteristics between the acidic and neutral/alkaline springs in the Rehai geothermal area are a direct reflection of their various geological geneses. The DRTY-02 and DRTY-08 springs had low pH values (2.78~3.04) and low Cl^−^ but high SO_4_^2−^ concentrations and belong to typical steam-heated acid springs. This is the result of the heating of shallow groundwater by geothermal steam separated from rapidly ascending deep geothermal fluids via adiabatic cooling. The acidic H_2_S originating from geothermal steam was oxidized to sulfuric acid in the near-surface oxidizing environment [48]. Compared with DRTY-02 and DRTY-08, the pH values and Cl^−^ concentrations of the WGQ spring and the ZZQ spring as well as the samples collected from its flow path were higher (2.81~5.52), and their SO_4_^2−^ concentrations were lower, indicating that their formation was the result of mixing of steam-heated acid water with neutral Cl/Na-rich water to varying degrees. The main dissolved components of the neutral/alkaline hot springs DGG, YJQ-R, HMZP-M, and HMZP-L (as well as the samples from their flow paths) were Na^+^, K^+^, and Cl^−^, with concentrations ranging from 244.4 to 807.5 mg/L, from 44.9 to 109.5 mg/L, and from 285.1 to 702.8 mg/L, respectively. These were much higher than those in the acidic hot springs. These neutral/alkaline hot springs are the discharge of deep geothermal fluids at the surface undergoing different cooling processes (adiabatic cooling, conduction cooling, mixing with shallow groundwater, or a combination of some of them) [49]. Controlled by their formation mechanisms, the tungsten concentrations (0.1–12.3 µg/L) in the acidic hot springs were higher than those in common natural waters (e.g., precipitation, seawater, and river water) but much lower (30.88–76.98 µg/L) than those in the neutral/alkaline hot springs. Another significant difference between the acidic and neutral/alkaline hot springs was that the tungsten concentration rapidly decreased along the flow path of the former but changed little along the flow path of the latter (Figure S3), in accordance with what Guo et al. [24] observed previously in Rehai. 

Contrary to the distribution of tungsten in the Rehai hot springs, the tungsten concentrations in the acidic hot spring sediments were significantly higher than those in the neutral/alkaline hot spring sediments. For example, the tungsten concentrations in the ZZQD1 spring (pH = 3.46) and its spring vent sediment were 12.0 µg/L and 991 µg/g, respectively, while these values were 30.9 µg/L and 160 µg/g and 68.3 µg/L and 11.0 µg/g for the HMZP-L spring (pH = 7.20) and the YJQ spring (pH = 9.15), respectively (Table S5). The distribution of tungsten in the hot spring sediments was also in accordance with the findings of Guo et al. [24].

### 3.2. Transformation of Tungstates to Polytungstates under Acidic Conditions as Well as Their UV-Vis Detection and Thermodynamic Simulation

**Figure 2:**
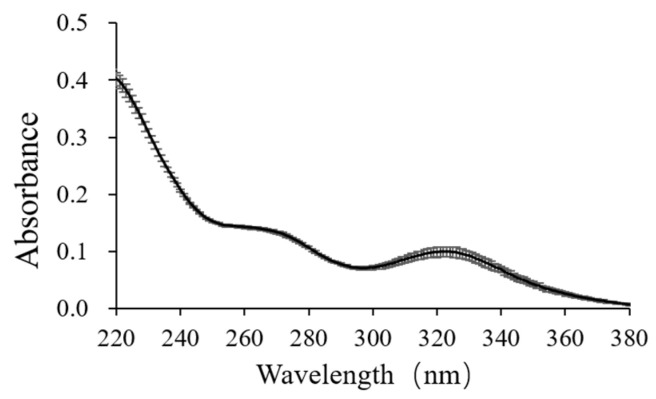
The absorbance at wavelengths of 220~380 nm after one-hour preparation of 100 µM Na_2_WO_4_·2H_2_O.

Previous studies demonstrated that hexameric and decameric tungstates have characteristic UV-Vis peaks at wavelengths of 275 and 320 nm, respectively [43]. Accordingly, the formation of polytungstates under specific conditions in this study was successfully identified. Specifically, the laboratory prepared 100 µmol/L Na_2_WO_4_·2H_2_O solution without pH adjustment or with pH adjustment to 9 which showed no characteristic peaks during UV-Vis detection, indicating that tungsten existed as monomeric tungstate in the solution. However, after acidification (pH = 3) for 1 h, characteristic peaks emerged at 275 and 320 nm (Figure 2), indicating that monomeric tungstate could rapidly convert to polymeric tungstates (hexameric and decameric tungstates) under acidic conditions. In addition, the absorbance at 275 nm increased gradually within 24 h after acidification, while the absorbance at 320 nm decreased correspondingly (Table 2), indicating that hexameric tungstate was more stable than decameric tungstate under acidic conditions.

Due to the matrix effect and the high detection limit of tungsten, UV-Vis spectrophotometry could not be used to identify the polytungstates in natural waters (including the hot springs investigated in this study). Unfortunately, so far there have been no other reliable methods for quantitative analysis of polytungstates in natural waters, and only analyses of polytungstates in laboratory-prepared solutions were available [50,51]. The identification of polytungstates using the UV-Vis method in this study demonstrated that an acidic condition does favor the formation of polytungstates and provided indirect evidence for the possible existence of polytungstates in the acidic hot springs in Rehai. Moreover, based on the chemical thermodynamic data of the monotungstate and polytungstates interconversion process (Table S2), the tungsten speciation in the Rehai hot springs was simulated (Table S6), revealing that polytungstates were commonly present in the acidic hot springs (mainly hexameric, decameric, and dodecameric tungstates) with proportions in total tungsten up to 93% but not in the neutral/alkaline hot springs.

### 3.3. Sorption of Tungstate and Polytungstate onto Goethite and Pyrite

#### 3.3.1. Sorption Kinetics

**Figure 3:**
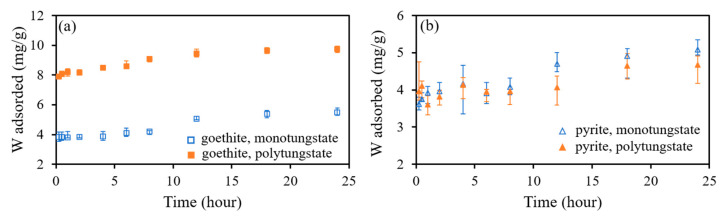
Adsorption of monotungstate and polytungstate onto goethite (**a**) and pyrite (**b**) as a function of contact time.

**Figure 4:**
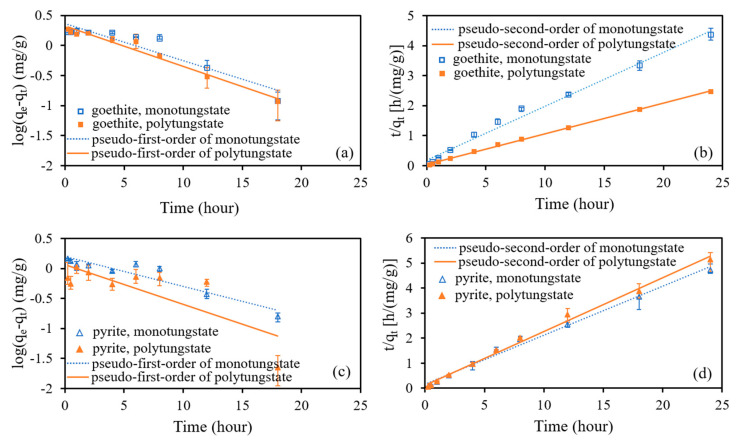
Pseudo-first-order (**a**) and pseudo-second-order (**b**) models for monotungstate and polytungstate adsorption on goethite. Pseudo-first-order (**c**) and pseudo-second-order (**d**) models for monotungstate and polytungstate adsorption on pyrite.

The results of the kinetics study of monotungstate and polytungstate adsorption onto goethite and pyrite are presented in Figure 3. Tungsten adsorbed onto goethite and pyrite increased rapidly within 1 h of the reaction and then slowly increased until equilibrium was reached at 18 h. Notably, polytungstate adsorbed onto goethite was significantly greater than that of the other three cases. Figure 4 shows the fitting results of the pseudo-first-order and pseudo-second-order kinetic models, and the fitted parameter values of the models are provided in Table 3. The pseudo-second-order kinetic model provided a better description of the experimental data than the pseudo-first-order kinetic model did, as shown by the higher determination coefficients of the former (R^2^ = 0.989–0.999 against 0.616–0.972). In addition, the calculated *q_e,cal_* was closer to the experimental *q_e_* using the pseudo-second-order kinetic model than that using the pseudo-first-order kinetic model. For example, the amounts of monotungstate adsorbed onto goethite at adsorption equilibrium were 5.50, 2.30, and 5.59 mg/g according to the experiment results, the pseudo-first-order model, and the pseudo-second-order model, respectively. This indicates that the adsorption of both monotungstate and polytungstate onto goethite and pyrite was controlled mainly by the chemisorption process. 

#### 3.3.2. Sorption Isotherms

**Figure 5:**
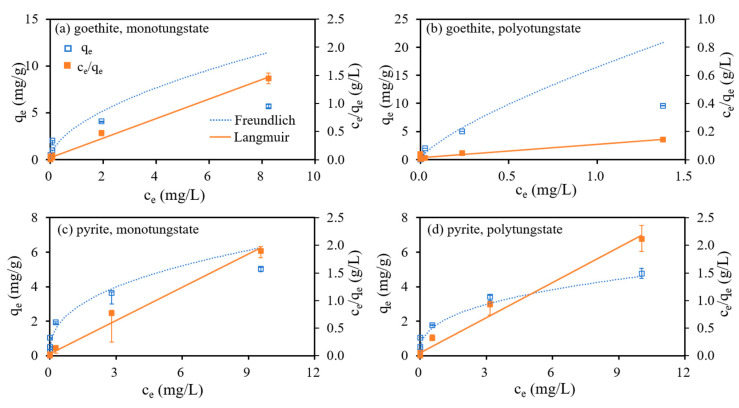
Freundlich and Langmuir isotherm models for monotungstate and polytungstate adsorption onto goethite and pyrite.

The fitting results of the isothermal adsorption of monotungstate and polytungstate onto goethite and pyrite using the Freundlich and Langmuir models are presented in Figure 5 and Table 4. Compared to the Freundlich model, the Langmuir model was more suitable to fit the experimental data (R^2^ = 0.981–0.992 against 0.557–0.866). The Langmuir model indicated *q_m_* in the descending order of 10.47 mg/g for polytungstate adsorption onto goethite, 5.57 mg/g for monotungstate adsorption onto goethite, 4.97 mg/g for monotungstate adsorption onto pyrite, and 4.70 mg/g for polytungstate adsorption onto pyrite.

#### 3.3.3. Effects of Temperature and Ionic Strength on Adsorption

**Figure 6:**
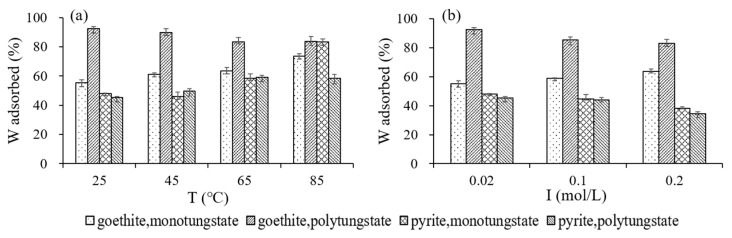
Effects of temperature (**a**) and ionic strength (**b**) on the adsorption of tungsten by pyrite and goethite. T: temperature; I: ironic strength; W: tungsten.

The effects of temperature and ionic strength on monotungstate or polytungstate adsorption onto goethite or pyrite varied to some degree (Figure 6). On the one hand, the adsorption rate (R) of monotungstate and polytungstate onto pyrite increased with increasing reaction temperature (Figure 6a); when the reaction temperature was elevated from 25 to 85 °C, the R raised from 48 to 84% and from 45 to 58% for monotungstate and polytungstate, respectively. The adsorption of monotungstate onto goethite was consistent with that onto pyrite when the reaction temperature increased (the R raised from 55 to 73%). However, the goethite showed a slightly lower affinity with polytungstate at elevated temperatures (the R decreased from 93 to 84%). On the other hand, the increase in ionic strength inhibited the adsorption of monotungstate and polytungstate onto pyrite (Figure 6b); when the NaCl concentration increased from 0.02 to 0.2 mol/L, the R decreased by 10 and 11% for monotungstate and polytungstate, respectively. Nevertheless, the effect of ionic strength on the adsorption of monotungstate and polytungstate onto goethite was variable; the R of monotungstate tended to increase (from 55 to 64%) with the increase in ionic strength from 0.02 to 0.2 mol/L, while the corresponding R of polytungstate showed a slightly decreasing tendency (from 93 to 83%). 

#### 3.3.4. Characterization of the Solid Samples before and after Tungsten Sorption

The XRD patterns of the solid samples reacted with monotungstate and polytungstate still retained the characteristic peaks of pristine goethite and pyrite (Figure S4). In addition, new diffraction peaks at 31.8°, 45.6°, 66.5°, 75.6°, and 84.4° appeared due to the crystallized NaCl which was added to the solutions to adjust their ionic strengths. These results suggest that the decrease in tungsten concentration in the solutions was primarily associated with the adsorption of tungsten onto goethite or pyrite without inducing precipitation of any tungsten-bearing minerals during the adsorption process. 

The goethite had elongated and needle-like crystals that were loosely aggregated and porous with an uneven surface, as shown in the SEM image (Figure S5a). The overall irregular porous structure of the crystals with a large specific surface area provides necessary channels and sufficient space for tungsten adsorption. The interspaces between the goethite crystals were filled via adsorption of tungsten that, in turn, became slightly rounded and short columns and tended to be generally smooth (Figure S5b,c). In addition, the atomic proportions of tungsten in the solid samples after adsorbing monotungstate and polytungstate reached 0.18 and 0.31%, respectively, according to the EDX analysis (Table S7), thus validating that tungsten had indeed been adsorbed onto goethite. The crystals of pristine pyrite were plate-like with a flat and smooth surface, while attached particles appeared on the surface and in the interspaces after adsorption (Figure S5d–f). Moreover, the atomic proportions of tungsten in the pyrite samples reacted with monotungstate and polytungstate were 0.18 and 0.12%, respectively, also verifying that tungsten had been adsorbed. 

**Figure 7:**
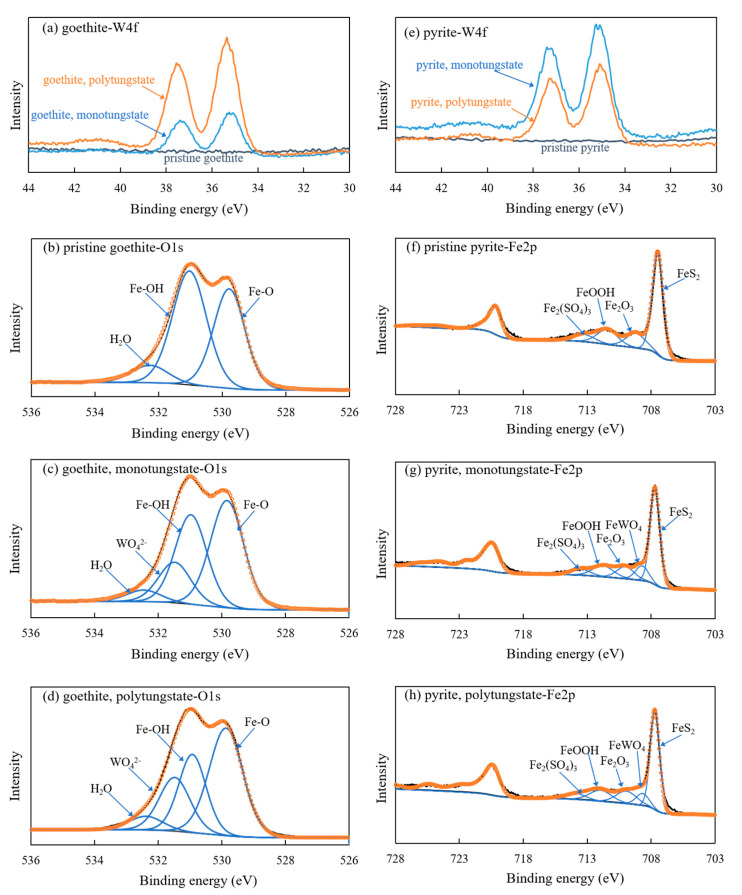
XPS spectra of W4f, O1s, and Fe2p for pristine and mono/polytungstate-loaded goethite and pyrite samples.

The W4f XPS spectra of the reacted goethite and pyrite samples recorded two new peaks at 35.2 and 37.4 eV corresponding to the W4f_7/2_ (35.1 eV, [52]) and W4f_5/2_ (37.7 eV, [53]) peaks of Na_2_WO_4_, respectively (Figure 7a,e), also validating the adsorption of tungsten onto goethite and pyrite.

## 4. Discussion

As mentioned earlier, it was observed in our previous study [24] that geothermal tungsten in Rehai tends to accumulate in iron-rich hot spring sediments. This was evidenced by a rough positive relation between iron and tungsten in the sediments. Further inspection indicated that the transfer of geothermal tungsten from hot springs to iron-rich sediments was controlled primarily by the sorption of tungsten to iron-bearing minerals, typically goethite and pyrite, as there were no tungsten-bearing minerals found in the sediments, and the Rehai hot springs were generally undersaturated with respect to these minerals, such as tungstenite. However, there is still a lack of understanding of the critical factors affecting the sorption of geothermal tungsten to hot spring sediments. That is, a number of key issues here need to be further studied—e.g., which iron-bearing mineral in the sediments is capable of adsorbing the most geothermal tungsten? Which hydrochemical parameters in the hot springs are most critical to tungsten sorption by iron-bearing minerals? How does the speciation of tungsten in the hot springs affect its transfer to hot spring sediments? A detailed discussion is given below.

### 4.1. Effects of Iron-Bearing Mineral Type on Tungsten Sorption to Hot Spring Sediment

The enrichment of tungsten in hot spring sediments is significantly influenced by the types of iron-bearing minerals in the sediments, which are crucial for the adsorption of geothermal tungsten. In Rehai, extreme enrichment of tungsten was more likely to occur in sediments rich in iron oxide minerals than in those rich in iron sulfide minerals. That is, the low iron concentration in the hot spring sediments resulted in low tungsten concentration, and with similar iron concentrations, sediments containing primarily iron oxide minerals have much higher tungsten concentrations than those containing primarily iron sulfide minerals. Typical examples in the study area were the HMZP-L (pH = 7.20) and HMZP-M (pH = 7.88) springs, with aqueous tungsten (all existing as monotungstate) concentrations of 30.88 and 40.59 µg/L, respectively. The iron concentrations in the corresponding sediment samples had close values of 61.3 and 47.3 mg/g for HMZP-L and HMZP-M, respectively. However, the HMZP-L sediment rich in goethite (Figure S6) had a much higher tungsten concentration than the HMZP-M sediment rich in pyrite (Figure S6) (160 µg/g against 7.3 µg/g). In contrast, another hot spring (i.e., DGG) in the study area with similar pH (7.90) but a higher tungsten concentration (77.98 µg/L; all existing as tungstate) had a very low tungsten concentration in the sediment (0.3 µg/g) because of the low iron concentration in the sediment (0.2 mg/g; depleted of both iron oxide minerals and iron sulfide minerals). 

As previously mentioned, the conducted adsorption experiment verified that goethite (a typical representative of iron oxide minerals) has a higher affinity to tungsten compared with pyrite (a typical representative of iron sulfide minerals). To understand the mechanisms responsible for the greater tungsten adsorption capacity of goethite than pyrite, the solid samples before and after tungsten adsorption were analyzed by XPS (Figure 7). The Fe-O bond (529.8 eV, [54]), Fe-OH bond (531.1 eV, [54]), and H_2_O (532.3 eV, [55]) were present in the O1s spectra of the unreacted goethite sample. After adsorption of monotungstate and polytungstate, WO_4_^2−^ (531.3 eV, [53]) appeared in both samples and accounted for 16.3 and 21.3% of the total oxygen atoms, respectively, while the proportions of the O atoms in the Fe-OH bond decreased from 42.9 to 34.8 and 27.1%, respectively (Table 5). As to unreacted pyrite, the Fe2p3/2 spectra exhibited an FeS_2_ (707.3 eV, [56]) peak accompanying a small amount of air oxidation products such as Fe_2_O_3_ (709.9 eV, [57]), FeOOH (711.7 eV, [55]), and Fe_2_(SO_4_)_3_ (713.3 eV, [58]). Similarly, an FeWO_4_ (709.2 eV, referring to FeO binding energy, [55]) peak occurred in the Fe2p3/2 spectra of the pyrite samples reacted with monotungstate and polytungstate, and its proportions in the total Fe atoms were 11.2 and 9.3%, respectively, while the proportions of Fe atoms in FeS_2_ reduced by 5.4 and 6.7%, respectively (Figure 7g,h and Table 6). Therefore, it is reasonable to speculate that iron atoms linked to -OH and -S in goethite and pyrite comprise the active sites for tungsten adsorption. In addition, goethite has more active sites than pyrite in view of the larger changes in the proportion of Fe-OH in the O1s spectrum of goethite than that of FeS_2_ in the Fe2p3/2 spectrum of pyrite upon tungsten adsorption, leading to a stronger tungsten adsorption capability of goethite than pyrite. 

### 4.2. Effects of Aqueous Environment Parameters on Tungsten Sorption to Hot Spring Sediment

From the hydrochemical characteristics of the Rehai hot springs and the tungsten concentrations in their corresponding sediments, it can be found that of the various aqueous environmental parameters, pH was the most important in terms of controlling the tungsten concentration in sediment. Specifically, the sediment samples with the highest tungsten contents (71.0–991 µg/g) occurred in the outflow channel of an acidic hot spring (i.e., the ZZQ spring outflow channel; pH = 2.81–3.29) that had much lower tungsten concentrations than the neutral/alkaline hot springs. Geochemical simulations (Table S6) indicated that polytungstates were the main tungsten species in the ZZQ spring [24]. This is in accordance with the insight obtained from the laboratory experiments of this study that transformation of monotungstate to polytungstates is favored under acidic conditions. In addition, the sediments in the ZZQ spring outflow channel were iron-rich, with goethite as the major iron-bearing mineral. Combined with the knowledge that the adsorption capacity of goethite for polytungstate is much stronger than that of goethite for monotungstate as well as that of pyrite for both monotungstate and polytungstate, it is safe to conclude that the formation of polytungstates in the hot springs and their strong adsorption onto the goethite in the sediments are the controlling factors leading to the extreme enrichment of tungsten in the sediments. 

In contrast, although the neutral/alkaline hot springs had much higher aqueous tungsten concentrations and some of their corresponding sediments (e.g., HMZP-L) were goethite-rich as well, the tungsten concentrations in these sediments were still significantly lower than those in the ZZQ spring outflow channel. This resulted from the fact that all the tungsten in the neutral/alkaline hot springs existed as monotungstate rather than polytungstates. However, the iron-rich sediments in the acidic hot spring vents (e.g., DRTY−02 and WGQ with pH and Fe concentration in sediment of 2.78 and 15.0 mg/g and 5.52 and 19.9 mg/g, respectively) did not show significant adsorption of aqueous tungsten either (W concentrations in sediment being 8.1 and 16.1 µg/g, respectively). In fact, although these sediments were iron-rich, only pyrite was observed using SEM. Hence, it is presumed that the iron-bearing minerals in them consist mainly of iron sulfide minerals with low tungsten adsorption capacity. Moreover, the extremely low total tungsten concentrations in DRTY-02 and WGQ (0.30 and 0.51 µg/L, respectively) implied that they should have very low polytungstate concentrations as well, though tungsten exists primarily as polytungstates under acidic conditions that can be more easily adsorbed than monotungstate. In summary, the lack of tungsten in the “source” directly caused the low tungsten concentrations in the “sink”. Thus, it is speculated that there may be a lower limit of total tungsten concentration in hot springs for the enrichment of tungsten in sediment. Once the total aqueous tungsten concentrations were below this critical value, the influence of the above-mentioned controlling parameters (e.g., pH, aqueous tungsten speciation, Fe concentration in sediment, and type of iron-bearing minerals) would no longer be significant. When the total aqueous tungsten concentrations were above the critical value, however, the total tungsten concentration’s effect on the transport of aqueous tungsten to the sediment would be much smaller than that of the tungsten speciation in a hot spring. According to the total tungsten concentrations in the ZZQ, DRTY-02, and WGQ springs, the critical value should be much lower than 10 µg/L but somewhat higher than 0.5 µg/L. Except for pH and critical total aqueous tungsten concentration, other aqueous environment parameters had limited effects on the tungsten adsorption onto the hot spring sediments. As presented previously, there were no significant correlations between the tungsten concentrations in the hot spring sediments and the temperature, ionic strength, and hydrochemical type of the hot spring. Controlled laboratory experiments also showed that temperature and ionic strength variations did not change the tungsten adsorption capacities of goethite and pyrite to a large extent.

## 5. Conclusions

Transport of aqueous tungsten to hot spring sediment is one of the key processes for the release of geothermal-system-derived tungsten to the surface environment that is an important part of the global geochemical cycle of tungsten. This study has indicated that the requirements for the substantial transport of aqueous tungsten to hot spring sediment include total aqueous tungsten concentration not below a certain threshold, formation of polytungstates under acidic conditions, and enrichment of iron oxide minerals represented by goethite in sediment. The co-occurrence of these critical geochemical conditions could ensure an extreme enrichment of tungsten in hot spring sediments. However, a lack of any of these conditions would result in limited tungsten adsorption and relatively low tungsten concentrations in the sediments. 

The above conclusions are robustly supported by the geochemical investigations of the Rehai hot springs and their corresponding sediments as well as the results of systematic laboratory experiments of tungsten adsorption onto typical iron-bearing minerals and transformation of monotungstate to polytungstates. In summary, for acidic hot springs with aqueous tungsten concentrations that are not too low (i.e., not much less than 10 µg/L referring to the tungsten concentration in the ZZQ spring), toxic tungsten would be effectively immobilized within the geothermal areas if the hot spring sediments at the spring vents and the outflow channels are iron-rich and goethite-dominant. Otherwise, aqueous tungsten in the hot springs would have relatively high mobility and would be expected to transport with the hot spring waters all the way downstream, possibly further polluting the other natural waters in and around the hot spring area. In the Yunnan–Sichuan–Tibet Geothermal Province (YST) of China, the number and the flow rates of neutral/alkaline hot springs in the high-temperature hydrothermal area of Rehai were generally much higher than those of acidic hot springs. Moreover, it is common in YST that the rivers flowing through a geothermal area and receiving the hot spring waters serve as the local drinking water sources. Typical examples are the Zaotang River flowing through the Rehai geothermal area [49], the Zangbo River through the Yangbajain geothermal area [59], the Luolang River through the Yangyi geothermal area [60], the Changma River through the Daggyai geothermal area [24], and the Xiangbai River through the Banglazhang geothermal area [61]. Therefore, the negative environmental effects of tungsten derived from high-temperature geothermal systems in China and worldwide cannot be ignored and should be further studied.

## Figures and Tables

**Table 1:** In situ parameters and the concentrations of Fe and W in the Rehai hot springs. The data for ZZQ, ZZQ-D1, ZZQ-D2, ZZD-D3, ZZQ-D4, and ZZQ-D5 are from Guo et al. [29].

Sample	T/°C	pH	EC (µs/cm)	Sulfide (mg/L)	W (µg/L)	Fe (mg/L)
DRTY-02	49	2.78	815	n.d.	0.30	4.48
DRTY-08	79	3.04	902	105	0.10	0.41
ZZQ	89	2.81	600	0.04	12.3	0.94
ZZQD1	73	3.46	557	n.d.	12.0	0.76
ZZQD2	47	3.62	569	n.d.	10.72	0.74
ZZQD3	41	3.30	541	n.d.	7.50	0.53
ZZQD4	39	3.21	553	n.d.	6.91	0.65
ZZQD5	38	3.29	568	n.d.	5.65	0.50
WGQ	41	5.52	398	1.40	0.51	0.52
HMZP-L	66	7.20	1680	0.00	30.88	1.65
HMZP-LD1	60	7.52	1890	0.02	34.36	1.01
HMZP-LD2	38	7.56	1891	n.d.	36.50	0.80
HMZP-LD3	29	8.12	1885	n.d.	34.02	0.63
HMZP-M	77	7.88	1874	0.10	40.59	0.39
DGG	84	7.90	4197	0.17	76.98	0.08
YJQ-R	90	9.15	3627	4.20	68.26	0.09

**Table 2:** Variation in the absorbance of 100 µM Na_2_WO_4_·2H_2_O (pH = 3) with reaction time.

Time	275 nm	320 nm
1 h	0.122	0.098
6 h	0.126	0.09
12 h	0.127	0.083
24 h	0.134	0.052

**Table 3:** Kinetics constants for tungsten sorption onto goethite and pyrite.

Sample	*c_e_*	*q_e_*	Pseudo-First-Order Kinetics			Pseudo-Second-Order Kinetics		
	(mg/L)	(mg/g)	*k*_1_	*q_e,cal_*	R^2^	*k*_2_	*q_e,cal_*	R^2^
Goethite, monotungstate	8.61	5.50	0.14	2.30	0.877	0.168	5.59	0.989
Goethite, polytungstate	1.04	9.75	0.15	2.11	0.972	0.264	9.81	0.999
Pyrite, monotungstate	9.46	5.08	0.11	1.57	0.884	0.26	5.09	0.993
Pyrite, polytungstate	10.18	4.67	0.15	1.15	0.616	0.38	4.67	0.994

**Table 4:** Isotherm constants for tungsten sorption onto goethite and pyrite.

Sample	Langmuir Model			Freundlich Model		
	*K_L_*	*q_max_*	R^2^	*K_F_*	*n*	R^2^
Goethite, monotungstate	5.21	5.75	0.992	3.55	1.80	0.846
Goethite, polytungstate	6.82	10.47	0.929	16.46	1.35	0.866
Pyrite, monotungstate	6.27	4.97	0.988	2.59	2.56	0.676
Pyrite, polytungstate	4.20	4.70	0.981	2.00	2.76	0.557

**Table 5:** Fitting parameters for O1s spectra of pristine and mono/polytungstate-loaded goethite.

Sample	Peak	Energy (eV)	FWHW (eV)	Percent (%)
Pristine goethite	O^2−^	529.8	1.35	50.5
	OH^−^	531.1	1.30	42.9
	H_2_O	532.4	1.30	6.6
Goethite, monotungstate	O^2−^	529.8	1.30	44.1
	OH^−^	531.0	1.22	34.8
	WO_4_^2−^	531.5	1.25	16.3
	H_2_O	532.4	1.30	4.8
Goethite, polytungstate	O^2−^	529.8	1.30	45.9
	OH^−^	531.0	1.25	27.1
	WO_4_^2−^	531.5	1.19	21.3
	H_2_O	532.4	1.29	5.7

**Table 6:** Fitting parameters for Fe2p3/2 spectra of pristine and mono/polytungstate-loaded pyrite.

Sample	Peak	Energy (eV)	FWHW (eV)	Percent (%)
Pristine pyrite	FeS_2_	707.4	1.09	61.3
	Fe_2_O_3_	709.6	1.88	14.7
	FeOOH	711.4	1.95	16.0
	Fe_2_(SO_4_)_3_	713.3	2.04	8.0
Pyrite, monotungstate	FeS_2_	707.6	0.94	55.9
	FeWO_4_	708.9	1.10	11.2
	Fe_2_O_3_	709.9	1.84	12.3
	FeOOH	711.7	1.91	13.4
	Fe_2_(SO_4_)_3_	713.6	1.95	7.3
Pyrite, polytungstate	FeS_2_	707.6	0.99	54.6
	FeWO_4_	708.9	1.05	9.3
	Fe_2_O_3_	709.9	1.87	14.8
	FeOOH	711.9	1.95	14.2
	Fe_2_(SO_4_)_3_	713.6	1.92	7.1

## Data Availability

The data presented in this study are available on request from the corresponding author. The data are not publicly available due to the undergoing study of a parter in research group.

## Supplementary Materials

The following are available online at https://www.mdpi.com/article/10.3390/ijerph182312629/s1, Figure S1: Piper diagram of the Rehai hot springs; Figure S2: The XRD patterns of laboratory synthetic goethite (a) and pyrite flushed by HCl (b) compared with standard reference data for goethite and pyrite, respectively; Figure S3: Variation of tungsten and iron contents of ZZQ (a) and HMZP-L (b) springs and their flow paths (data of ZZQ spring and its flow path from Guo et al. [24]); Figure S4: The XRD results of pyrite and goethite before and after adsorbing the tungstate and polytungstate (Initial tungsten concentration = 100 μmol/L); Figure S5: The SEM results of pyrite and goethite before and after adsorbing the tungstate and polytungstate; Figure S6: The SEM-EDX results of WGQ, HMZP-M, HMZP-L, and ZZQ hot spring sediments in Rehai. The data of WGQ and HMZP-L are from Guo et al. [24]. Table S1: The limits of detection (LODs) and the limits of quantification (LOQs) for the constituents are in mg/L except for tungsten in μg/L. <LOD: not detected (n.d.). Table S2: Chemical thermodynamic data of varied tungsten species in aqueous solution; Table S3: Inductively coupled plasma mass spectrometry analyses of national rock standards in China; Table S4: Hydrochemical compositions of the hot springs in Rehai (in mg/L). The data of ZZQ, ZZQ-D1, ZZQ-D2, ZZD-D3, ZZQ-D4, and ZZQ-D5 are from Guo et al. [24]. Table S5: Concentrations of tungsten and iron in the sediments from the vents and the outflow channels of the Rehai hot springs (in mg/kg). The data are from Guo et al. [24], except for the WGQ spring. Table S6: Thermodynamically calculated tungsten speciation in the Rehai hot springs (in mol/L). The data of ZZQ, ZZQ-D1, ZZQ-D2, ZZD-D3, ZZQ-D4, and ZZQ-D5 are from Guo et al. [24]. Table S7: The EDX analysis results of pyrite and goethite before and after adsorbing the tungstate and polytungstate.

## References

[1] Dermatas D., Braida W., Christodoulatos C., Strigul N., Panikov N., Los M., Larson S. (2004). Solubility, sorption, and soil respiration effects of tungsten and tungsten alloys. Environ. Forensics.

[2] Koutsospyros A., Braida W.J., Christodoulatos C., Dermatas D., Strigul N.S. (2006). A review of tungsten: From environmental obscurity to scrutiny. J. Hazard. Mater..

[3] Strigul N.S., Koutsospyros A., Christodoulatos C. (2009). Tungsten in the former Soviet Union: Review of environmental regulations and related research. Land Contam. Reclam..

[4] Thomas V.G., Roberts M.J., Harrison P.T.C. (2009). Assessment of the environmental toxicity and carcinogenicity of tungsten-based shot. Ecotoxicol. Environ. Saf..

[5] USGS (2001). Water Quality Investigation Related to the Leukemia Cluster Fallon, Nevada.

[6] Sheppard P.R., Ridenour G., Speakman R.J., Witten M.L. (2006). Elevated tungsten and cobalt in airborne particulates in Fallon, Nevada: Possible implications for the childhood leukemia cluster. Appl. Geochem..

[7] Steinburg K.K., Relling M.V., Gallagher M.L., Greene C.N., Rubin C.S., French D., Holmes A.K., Carroll W.L., Koontz D.A., Sampson E.J. (2007). Genetic studies of a cluster of acute lymphoblastic leukemia cases in Churchill County, Nevada. Environ. Health Perspect..

[8] Tyrrell J., Galloway T.S., Abo-Zaid G., Melzer D., Depledge M.H., Osborne N.J. (2013). High urinary tungsten concentration is associated with stroke in the National Health and Nutrition Examination Survey 1999–2010. PLoS ONE.

[9] Pardus M.J., Sueker J.K. (2009). Occurrence and geochemistry of tungsten in the Carson River Basin, Nevada, USA. Land Contam. Reclam..

[10] Strigul N., Galdun C., Vaccari L., Ryan T., Braida W., Christodoulatos C. (2009). Influence of speciation on tungsten toxicity. Desalination.

[11] Strigul N., Koutsispyros A., Christodoulatos C. (2010). Tungsten speciation and toxicity: Acute toxicity of mono- and poly-tungstates to fish. Ecotoxicol. Environ. Saf..

[12] Strigul N. (2010). Does speciation matter for tungsten ecotoxicology?. Ecotoxicol. Environ. Saf..

[13] Kelly A.D.R., Lemaire M., Young Y.K., Eustache J.H., Guilbert C., Molina M.F., Mann K.K. (2013). In Vivo tungsten exposure alters B-cell development and increases DNA damage in murine bone marrow. Toxicol. Sci..

[14] Clausen J.L., Korte N. (2009). Environmental fate of tungsten from military use. Sci. Total Environ..

[15] Johannesson K.H., Dave H.B., Mohajerin T.J., Datta S. (2013). Controls on tungsten concentrations in groundwater flow systems: The role of adsorption, aquifer sediment Fe (III) oxide/oxyhydroxide content, and thiotungstate formation. Chem. Geol..

[16] Ertel W.H., O’Neill S.C., Dingwell D.B., Spettel B. (1996). Solubility of tungsten in a haplobasaltic melt as a function of temperature and oxygen fugacity. Geochim. Et Cosmochim. Acta.

[17] Wood S.A., Samson I.M. (2000). The hydrothermal geochemistry of tungsten in granitoid environments: I. Relative solubilities of ferberite and scheelite as a function of T, P, pH, and mNaCl. Econ. Geol..

[18] Schubert W.D., Lassner E., Walser P. Geology of Tungsten. Proceedings of the Itia Newsletter, International Tungsten Industry Association.

[19] Cui M., Johannesson K.H. (2017). Comparison of tungstate and tetrathiotungstate adsorption onto pyrite. Chem. Geol..

[20] Xu N., Christodoulatos C., Koutsospyros A., Braida W. (2009). Competitive sorption of tungstate, molybdate and phosphate mixtures onto goethite. Land Contam. Reclam..

[21] Bäckström M., Nilsson U., Häkansson K., Allard B., Karlsson S. (2003). Speciation of heavy metals in road runoff and roadside total deposition. Water Air Soil Pollut..

[22] Charter R.A., Tabatabai M.A., Schafer J.W. (1995). Arsenic, molybdenum, selenium, and tungsten contents of fertilizers and phosphate rocks. Commun. Soil Sci. Plant Anal..

[23] Fu M.H., Tabatabai M.A. (1988). Tungsten contents of soils, plants and sewage sludges in Iowa. J. Environ. Qual..

[24] Guo Q., Li Y., Luo L. (2019). Tungsten from typical magmatic hydrothermal systems in China and its environmental transport. Sci. Total Environ..

[25] Turekian K.K. (1968). Oceans.

[26] Kunzendorf H., Glasby G.P. (1992). Tungsten accumulation in Pacific ferromanganese deposits. Miner. Depos..

[27] Kishida K., Sohrin Y., Okamura K., Ishibashi J. (2004). Tungsten enriched in submarine hydrothermal fluids. Earth Planet. Sci. Lett..

[28] Hall G.E.M., Jefferson C.W., Michel F.A. (1988). Determination of W and Mo in natural spring waters by ICP-AES (inductively coupled plasma atomic emission spectrometry) and ICP-MS (inductively coupled plasma mass spectrometry): Application to South Nahanni river area N.W.T. J. Geochem. Explor..

[29] McCleskey R.B., Nordstrom D.K., Susong D.D., Ball J.W., Taylor H.E. (2010). Source and fate of inorganic solutes in the Gibbon River, Yellowstone National Park, Wyoming, USA. II. Trace element chemistry. J. Volcanol. Geotherm. Res..

[30] Krainov S.R. (1971). The influence of acidicalkaline conditions of underground waters upon the concentration and migration of rare elements in them. Geochem. Int..

[31] Planer-Friedrich B., Forberg J., Lohmayer R., Kerl C.F., Boeing F., Kaasalainen H., Stefansson A. (2020). Relative abundance of thiolated species of As, Mo, W, and Sb in hot springs of Yellowstone National Park and Iceland. Environ. Sci. Technol..

[32] Mohajerin T.J., Helz G.R., Johannesson K.H. (2016). Tungsten–molybdenum fractionation in estuarine environments. Geochim. Et Cosmochim. Acta.

[33] Sun J., Bostick B.C. (2015). Effects of tungstate polymerization on tungsten (VI) adsorption on ferrihydrite. Chem. Geol..

[34] Bednar A.J., Boyde R.E., Jones W.T., McGrath C.J., Johnson D.R., Chappell M.A., Ringelberg D.B. (2009). Investigation of tungsten mobility in soil using column tests. Chemosphere.

[35] Bai D., Liao Z., Zhao G., Wang X. (1994). The inference of magmatic heat source beneath the Rehai (hot sea) field of Tengchong from the result of magnetotelluric sounding. Chin. Sci. Bull..

[36] Govindaraju K. (1994). 1994 compilation of working values and sample description for 383 geostandards. Geostand. Geoanalytical Res..

[37] Hu Z., Gao S. (2008). Upper crustal abundances of trace elements: A revision and update. Chem. Geol..

[38] Appelo C.A.J., Postma D. (1996). Geochemistry, Groundwater, and Pollution.

[39] Parkhurst D.L., Appelo C.A.J. (1999). A Computer Program for Speciation, Batch-Reaction, One-Dimensional Transport, and Inverse Geochemical Calculations.

[40] Liao Z., Zhao P. (1999). Yunnan-Tibet Geothermal Belt—Geothermal Resources and Case Histories.

[41] Liu Y., Zong K., Kelemen P.B., Gao S. (2008). Geochemistry and magmatic history of eclogites and ultramafic rocks from the Chinese continental scientific drill hole: Subduction and ultrahigh-pressure metamorphism of lower crustal cumulates. Chem. Geol..

[42] Pope M.T. (1983). Heteropoly and Isopoly Oxometalates.

[43] Zhu S.S., Gu J.D. (1989). Interconversion Between Hexa- and Decatungstic Acids in Solutions. Chem. J. Chin. Univ..

[44] Lagergren S. (1898). About the theory of so-called adsorption of soluble substances. K. Sven. Vetensk. Handl..

[45] Ho Y.S., Mckay G. (1999). A kinetic study of dye sorption by biosorbent waste product pith. Resour. Conserv. Recycl..

[46] Freundlich H.M.F. (1906). Adsorption in solution. J. Phys. Chem..

[47] Foo K.Y., Hameed B.H. (2010). Insights into the modeling of adsorption isotherm systems. Chem. Eng. J..

[48] Guo Q., Liu M., Li J., Zhang X., Wang Y. (2014). Acid hot springs discharged from the Rehai hydrothermal system of the Tengchong volcanic area (China): Formed via magmatic fluid absorption or geothermal steam heating?. Bull. Volcanol..

[49] Guo Q.H., Wang Y.X. (2012). Geochemistry of hot springs in the Tengchong hydrothermal areas, Southwestern China. J. Volcanol. Geotherm. Res..

[50] Bednar A.J., Kirgan R.A., Johnson D.R., Russell A.L., Hayes C.A., McGrath C.J. (2009). Polytungstate analysis by SEC-ICP-MS and direct-infusion ESI-MS. Land Contam. Reclam..

[51] Bednar A.J., Mirecki J.E., Inouye L.S., Winfield L.E., Larson S.L., Ringelberg D.B. (2007). The determination of tungsten, molybdenum, and phosphorous oxyanions by HPLC-ICP-MS. Talanta.

[52] Ho S.F., Contarini S., Rabalais J.W. (1987). Ion-beam-induced chemical changes in the oxyanions (Moyn-) and oxides (Mox) where M = chromium, molybdenum, tungsten, vanadium, niobium and tantalum. J. Phys. Chem..

[53] Dadachova E., Mirzadeh S., Lambrecht R.M. (1995). Tungstate-Ion-Alumina Interation in a 188W.fwdarw. 188Re Biomedical Generator. J. Phys. Chem..

[54] Harvey D.T., Linton R.W. (1981). The Chemical Characterization of Hydrous Ferric Oxide by X-Ray Photoelectron Spectroscopy. Anal. Chem..

[55] McIntyre N.S., Zetaruk D.G. (1977). X-ray Photoelectron Spectroscopic Studies of Iron Oxides. Anal. Chem..

[56] Laajalehto K., Kartio I. (1992). XPS study of clean metal sulfide surfaces. Appl. Surf. Sci..

[57] Paparazzo E. (1987). XPS and auger spectroscopy studies on mixtures of the oxides SiO_2_, Al_2_O_3_, Fe_2_O_3_ and Cr_2_O_3_. J. Electron Spectrosc. Relat. Phenom..

[58] Brion D. (1980). Etude par spectroscopie de photoelectrons de la degradation superficielle de FeS2, CuFeS2, ZnS et PbS a l’air et dans l’eau. Appl. Surf. Sci..

[59] Guo Q.H., Wang Y.X., Liu W. (2007). Major hydrogeochemical processes in the two reservoirs of the Yangbajing geothermal field, Tibet, China. J. Volcanol. Geotherm. Res..

[60] Guo Q.H., Wang Y.X., Liu W. (2009). Hydrogeochemistry and environmental impact of geothermal waters from Yangyi of Tibet, China. J. Volcanol. Geotherm. Res..

[61] Guo Q.H., Liu M., Li J., Zhang X., Guo W., Wang Y. (2017). Fluid geochemical constraints on the heat source and reservoir temperature of the Banglazhang hydrothermal system, Yunnan-Tibet Geothermal Province, China. J. Geochem. Explor..

