# Supplementary material for: Tungsten Accumulation in Hot Spring Sediments Resulting from Preferred Sorption of Aqueous Polytungstates to Goethite
Source: Int J Environ Res Public Health. 2021 Nov 30;18(23):12629. doi: 10.3390/ijerph182312629 (PMC8656809; doi:10.3390/ijerph182312629)
Supplement: Supplementary file 1 [file ijerph-18-12629-s001.zip › ijerph-1444812-supplementary.pdf]

Appendix: supplementary data

S1. Preparation of Goethite and Pyrite

S1.1 Synthesis of Goethite

Referring to Schwertmann and Cornell [1], 180 mL of 5M KOH solution was added dropwise to 100 mL of 1M $FeNO_3 \cdot 9H_2O$ solution, and the mixture was diluted with ultrapure water to a final volume of 2 L, adjusted to pH=12 with HNO_3, and aged for more than 40 h at 85°C in an electric thermostatic oven. The slurry was centrifuged and washed with ultrapure water for several times until the conductivity of the supernatant was below 200 μs/cm. The solid was dried in an electric constant temperature drying oven, and then milled through a 100-mesh sieve and stored in a desiccator. The XRD analysis result was presented in Fig. S1a. The peak position, shape and relative intensity of the synthetic solid were consistent with those of the standard reference date of goethite. The SEM image presented a typical elongated needle shape and a good degree of crystallization (Fig. S5a), the main elements were O and Fe, and the O/Fe ratio = 2.19 (Table S7), which was close to the theoretical value of 2.0, indicating that the experimental synthesis product was goethite.

S1.2 Flushing of Pyrite Surface Oxides

Pyrite (purity>95 %, Wuhan Aopu) was firstly flushed with 0.1 M HCl for surface oxides, and then washed with ultrapure water. The flushed solid was vacuum dried and stored in a glove box until use, which was confirmed to still remain the characteristic of pyrite by XRD and SEM-EDX (see Fig. S1b, Fig. S5d and Table S7).

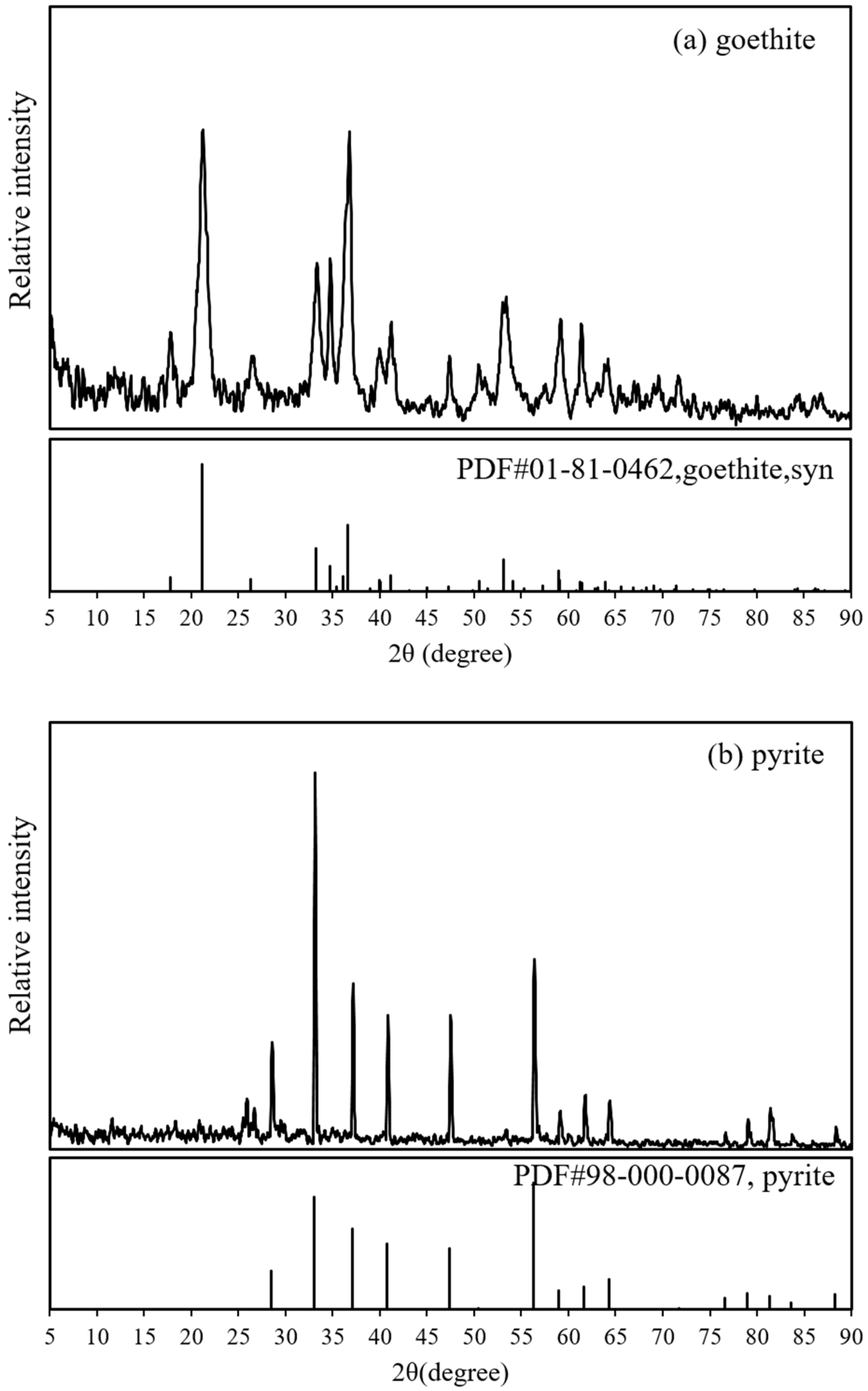

Figure S1. The XRD patterns of laboratory synthetic goethite (a) and pyrite flushed by HCl (b) compared with standard reference data for goethite and pyrite, respectively.

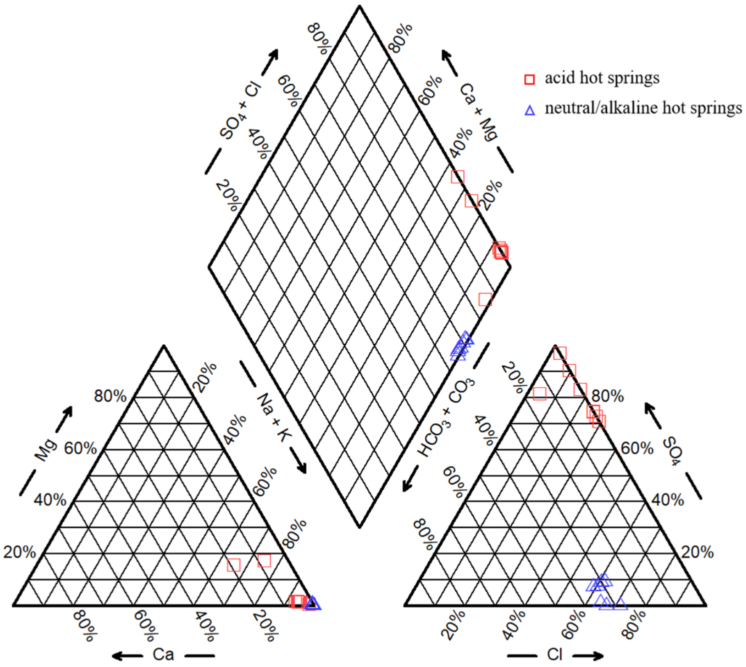

Figure S2. Piper diagram of the Rehai hot springs.

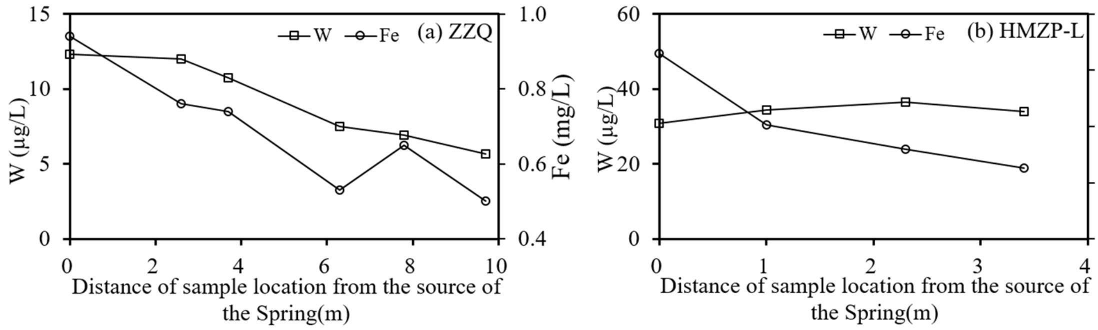

Figure S3. Variation of tungsten and iron contents of ZZQ (a) and HMZP-L (b) springs and their flow paths (the data of ZZQ spring and its flow path from Guo et al. [2]).

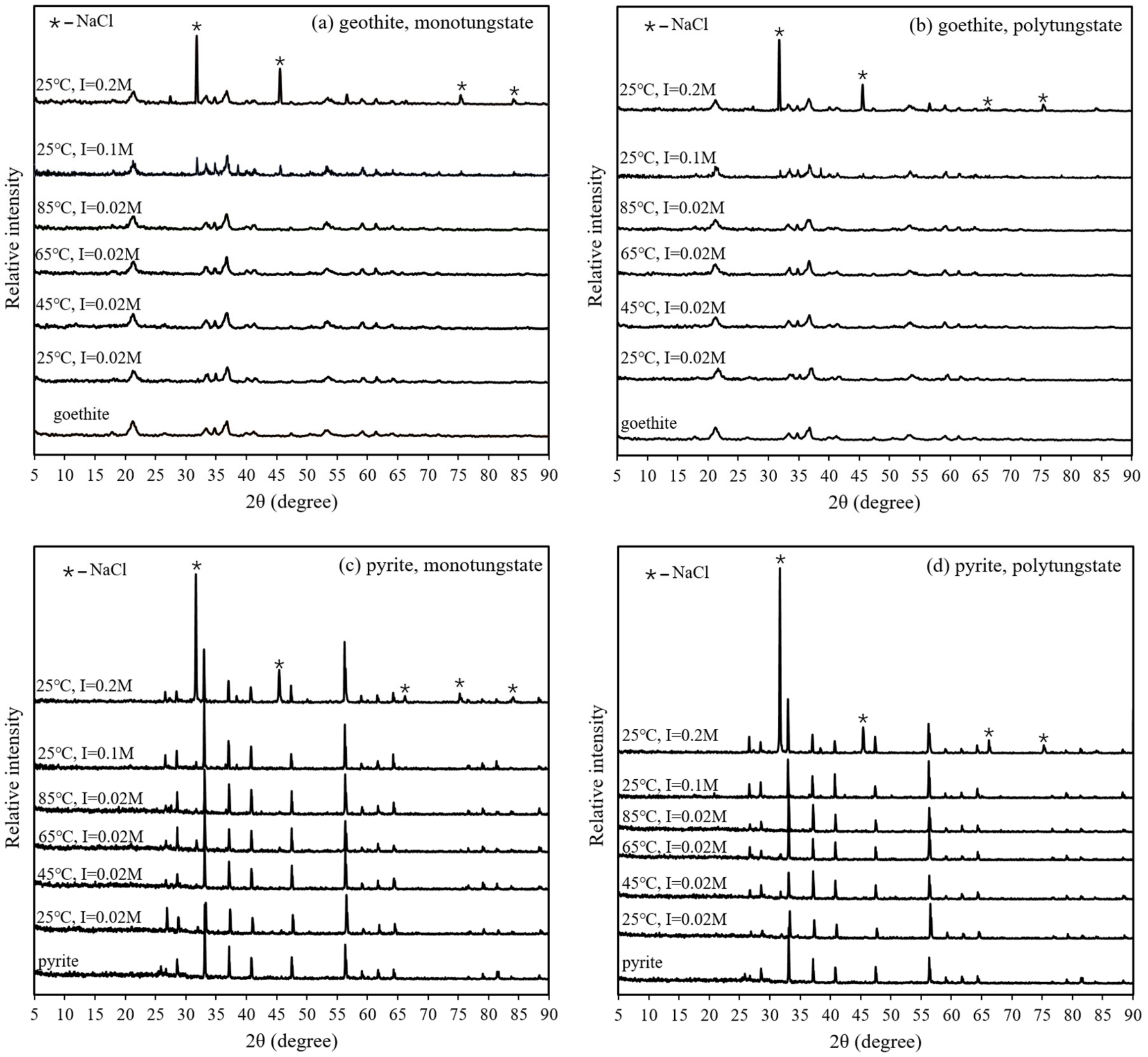

Figure S4. The XRD results of pyrite and goethite before and after adsorbing the monotungstate and polytungstate (Initial tungsten concentration = 100 μmol/L).

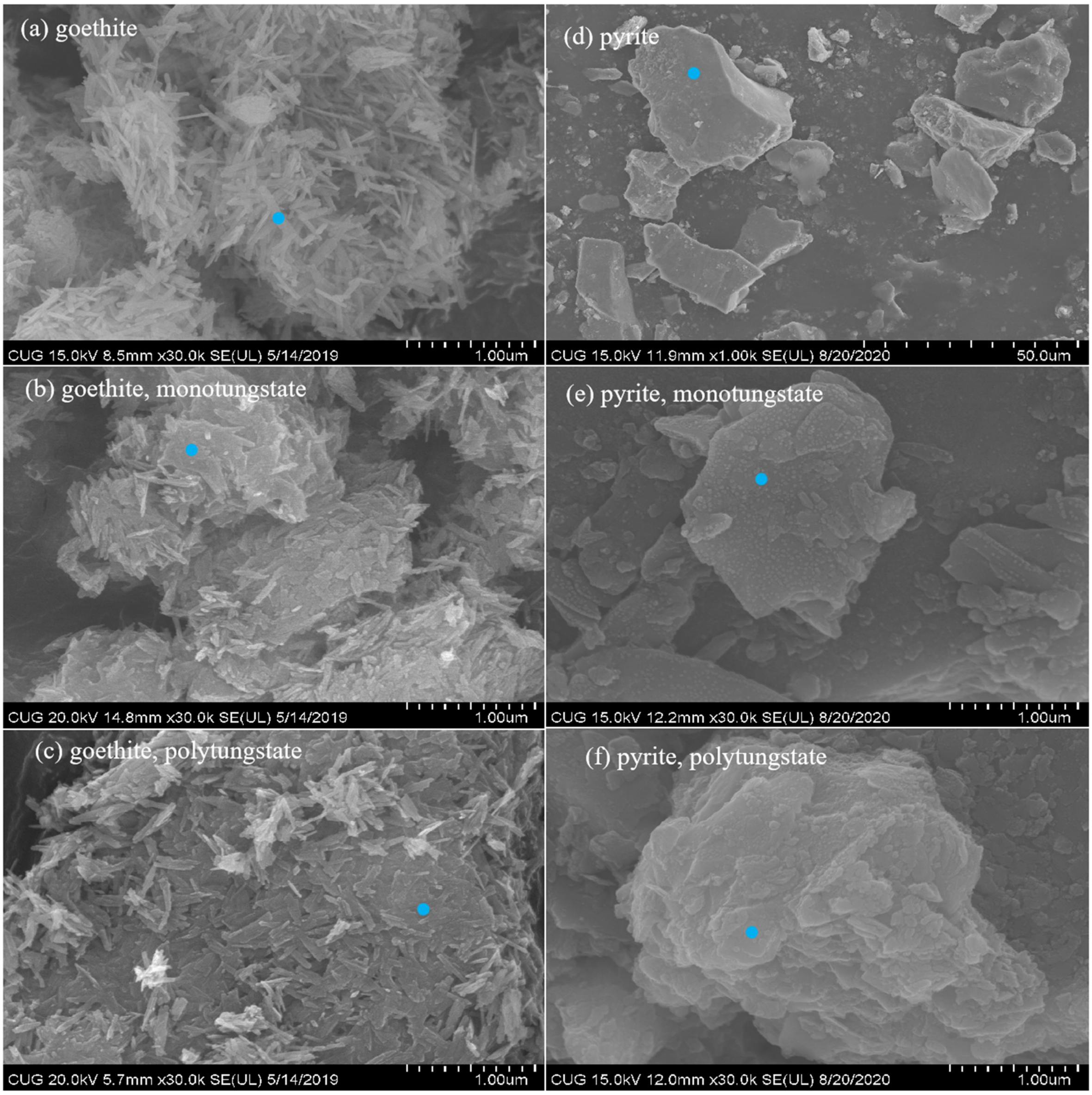

Figure S5. The SEM results of pyrite and goethite before and after adsorbing the monotungstate and polytungstate.

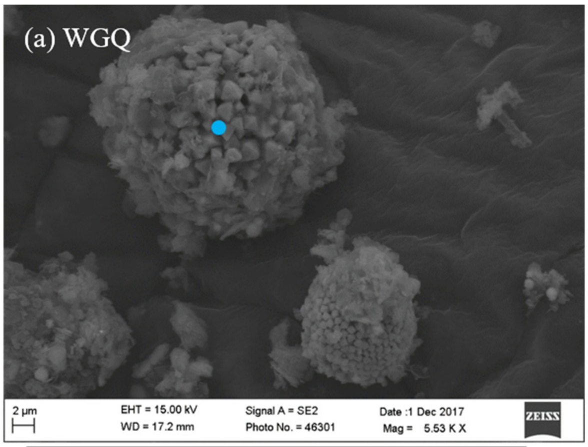

Weight percent (%)			
O	Al	S	Fe
9.2	0.5	50.9	39.4

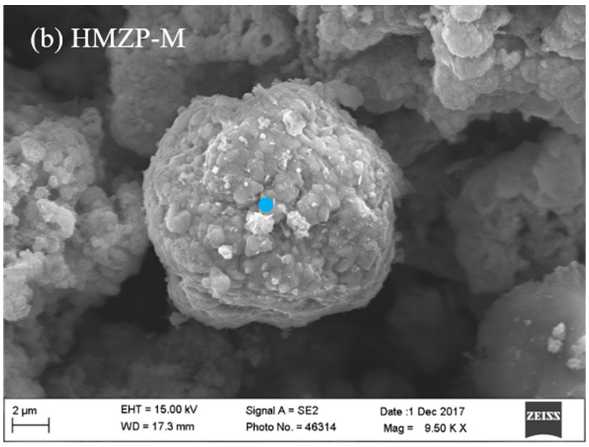

Weight percent (%)			
O	C	S	Fe
9.7	10.9	44.0	35.4

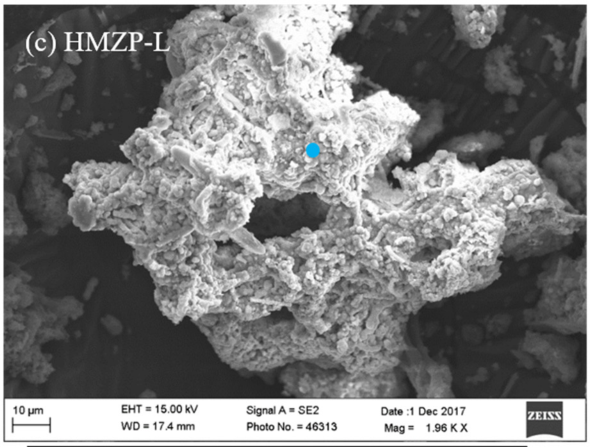

Weight percent (%)			
O	Si	As	Fe
33.8	7.8	2.5	55.9

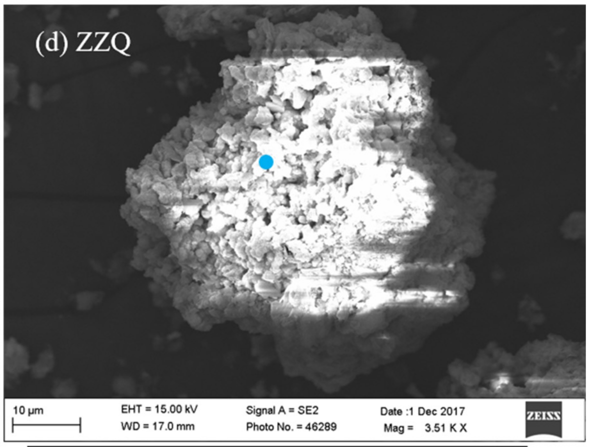

Weight percent (%)					
O	Si	Al	S	Fe	Zr
30.8	6.6	5.2	1.5	51.4	4.5

Figure S6. The SEM-EDX results of WGQ, HMZP-M, HMZP-L and ZZQ hot spring sediments in Rehai. The data of WGQ and HMZP-L were from Guo et al. [2].

Table S1

The limits of detection (LOD) and the limits of quantification (LOQ) for the constituents are in mg/L except for tungsten in μg/L. <LOD: not detected (n.d.).

Element	Limits of detection (LOD)[a]	Limits of quantification (LOQ)[b]
Sulfide	0.01	0.16
W	0.005	0.08
Fe	0.05	0.8
Alkalinity	0.3	1
SO_4	0.1	0.33
Cl	0.05	0.17
F	0.03	0.1
Na	0.08	6
K	0.08	6
Ca	0.02	1.6
Mg	0.03	0.5

a: The LOD was calculated as three times of the standard deviation of the concentration of each element in blank;

b: The LOQ was calculated by multiplying ten times of the standard deviation of the procedure blank concentration by the dilution factor (25 for Na, K, and Ca; 5 for W, Fe, Mg, and Sulfide; 1 for SO_4, Cl, F, and alkalinity).

Table S2. Chemical thermodynamic data of varied tungsten species in aqueous solution.

Species	Formula	Reaction	Log K	△H (KJ/mol)	Source
Tungstates	$H_nWO_4^{n-2}$	$WO_4^{2-} + H^+ = HWO_4^-$	3.60	45.3	[3]
	(n = 0, 1, 2)	$WO_4^{2-} + 2H^+ = H_2WO_4$	5.80	69.8	
Thiotungstates	WSO_3^{2-}	$WO_4^{2-} + H_2S = WSO_3^{2-} + H_2O$	3.08	--	[4]
	$WS_2O_2^{2-}$	$WSO_3^{2-} + H_2S = WS_2O_2^{2-} + H_2O$	3.22	--	
	WS_3O^{2-}	$WS_2O_2^{2-} + H_2S = WS_3O^{2-} + H_2O$	2.76	--	
	WS_4^{2-}	$WS_3O^{2-} + H_2S = WS_4^{2-} + H_2O$	2.36	--	
Polytungstates	$W_7O_{24}^{6-}$	$7WO_4^{2-} + 8H^+ = W_7O_{24}^{6-} + 4H_2O$	65.19	333	[5]
	$HW_7O_{24}^{5-}$	$7WO_4^{2-} + 9H^+ = HW_7O_{24}^{5-} + 4H_2O$	76.59	328	[6]
	$HW_6O_{21}^{5-}$	$6WO_4^{2-} + 7H^+ = HW_6O_{21}^{5-} + 3H_2O$	63.83	--	[3]
	$H_2W_6O_{22}^{6-}$	$6WO_4^{2-} + 6H^+ = H2W_6O_{22}^{6-} + 2H_2O$	53.68	231	[6]
	$H_2W_{12}O_{42}^{10-}$	$12WO_4^{2-} + 14H^+ = H2W_{12}O_{42}^{10-} + 6H_2O$	123.38	542	[6]
	$H_2W_{12}O_{40}^{6-}$	$12WO_4^{2-} + 18H^+ = H2W_{12}O_{40}^{6-} + 8H_2O$	149.59	--	[6]
	$W_{10}O_{32}^{4-}$	$10WO_4^{2-} + 16H^+ = W_{10}O_{32}^{4-} + 8H_2O$	129.63	--	[6]

Table S3. Inductively coupled plasma mass spectrometer analyses of national rock standards in China.

	Blank	GSD-9		GSD-10		GSD-12	
		Obt.	Ref.	Obt.	Ref.	Obt.	Ref.
Fe	0.01	15.1	15.3(±0.5)	0.5	0(±2.6)	12.1	11.9(±0.7)
W	0.00	1.78	1.8(±0.2)	1.57	1.6(±0.3)	35	37(±2)

In µg/g for W and blank, and mg/g for Fe.

Obt.: obtained values. Ref.: reference values.

The data after "±" are uncertainties, and the values in parentheses are reference values.

Table S4. Hydrochemical compositions of the hot springs in Rehai (in mg/L). The data of ZZQ, ZZQ-D1, ZZQ-D2, ZZD-D3, ZZQ-D4 and ZZQ-D5 were from Guo et al. [2].

Sample No.	HCO_3^-	CO_3^{2-}	SO_4^{2-}	Cl^-	F^-	Na	K	Ca	Mg
DRTY-02	0.00	0.00	226.9	5.6	1.5	3.6	8.9	2.20	1.11
DRTY-08	0.00	0.00	325.1	26.3	1.9	42.6	10.9	19.5	4.9
ZZQ	0.00	0.00	235.9	35.9	0.79	54.6	28.8	3.57	0.73
ZZQD1	0.00	0.00	165.1	41.6	1.38	63.9	31.6	2.84	0.65
ZZQD2	0.00	0.00	175.6	44.9	1.50	65.1	32.8	3.18	0.65
ZZQD3	0.00	0.00	145.8	40.5	1.27	57.8	27.1	2.76	0.66
ZZQD4	0.00	0.00	132.4	40.3	0.97	56.8	27.7	3.03	0.72
ZZQD5	0.00	0.00	139.0	42.7	1.20	58.0	27.7	2.95	0.67
WGQ	30.53	0.00	136.3	5.5	1.3	57.7	16.8	0.7	0.23
HMZP-L	238.07	0.78	0.0	285.1	7.6	244.4	44.9	0.9	0.88
HMZP-LD1	295.97	1.84	52.4	306.8	7.8	286.8	50.1	1.1	0.75
HMZP-LD2	251.30	1.04	63.7	317.0	7.9	285.6	50.0	0.9	0.83
HMZP-LD3	258.95	3.05	62.1	311.8	8.7	284.7	51.0	0.9	0.89
HMZP-M	256.05	4.78	50.2	291.3	8.2	265.3	43.9	0.9	0.52
DGG	586.56	27.82	18.0	702.8	17.8	807.5	109.5	0.7	0.29
YJQ-R	177.52	127.71	0.0	642.2	15.9	573.3	98.6	0.7	0.31

Table S5. Concentrations of tungsten and iron in the sediments from the vents and the outflow channels of the Rehai hot springs (in µg/g for W and blank, and mg/g for Fe). The data were from Guo et al. [2] except for the WGQ spring.

Sample	W	Fe	Sample	W	Fe
ZZQ	71.0	1.4	DRTY-02	8.1	15.0
ZZQD1	991	28.9	DRTY-08	16.0	0.7
ZZQD2	536	21.7	WGQ	16.1	19.9
ZZQD3	218	8.0	HMZP-L	160	61.3
ZZQD4	197	4.9	HMZP-M	7.3	47.3
YJQ-R	11.0	4.3	DGG	0.3	0.2

Table S6. Thermodynamically calculated tungsten speciation in the Rehai hot springs (in mol/L). The data of ZZQ, ZZQ-D1, ZZQ-D2, ZZD-D3, ZZQ-D4 and ZZQ-D5 were from Guo et al. [2].

Sample No.	Proportion of tungsten species in total tungsten			Thermodynamically calculated concentrations of monotungstates			Thermodynamically calculated concentrations of polytungstates							Thermodynamically calculated concentrations of thiotungstates			
	monotungstates	***polytungstates***	***thiotungstates***	WO_4^{2-}	HWO_4^{-}	H_2WO_4	$H_2W_{12}O_{40}^{6-}$	$H_2W_{12}O_{42}^{10-}$	$H_2W_6O_{22}^{6-}$	$HW_6O_{21}^{5-}$	$HW_7O_{24}^{5-}$	$W_{10}O_{32}^{4-}$	$W_7O_{24}^{6-}$	WO_3S^{2-}	$WO_2S_2^{2-}$	WOS_3^{2-}	WS_4^{2-}
DRTY-02	99.97%	0.03%	0.00%	2.20E-10	1.13E-09	2.74E-10	1.71E-17	2.92E-30	3.12E-21	2.92E-14	7.29E-17	5.13E-13	7.29E-17	0.00E+00	0.00E+00	0.00E+00	0.00E+00
DRTY-08	99.89%	0.02%	0.09%	5.59E-10	1.48E-09	1.92E-10	1.92E-17	1.50E-28	2.98E-20	1.20E-13	2.10E-16	2.28E-13	2.10E-16	2.04E-12	1.03E-14	1.79E-17	1.25E-20
ZZQ	7.25%	92.75%	0.00%	6.45E-10	2.96E-09	6.57E-10	1.62E-12	9.62E-25	1.55E-18	1.15E-11	6.88E-14	5.44E-09	5.24E-22	9.68E-13	2.01E-15	1.44E-18	4.13E-22
ZZQ-D1	23.67%	76.33%	0.00%	6.77E-09	7.13E-09	3.58E-10	6.43E-12	8.85E-22	2.37E-16	4.30E-10	1.40E-12	4.33E-09	4.35E-20	0.00E+00	0.00E+00	0.00E+00	0.00E+00
ZZQ-D2	38.24%	61.76%	0.00%	1.16E-08	8.53E-09	2.96E-10	5.67E-12	2.89E-21	6.42E-16	8.29E-10	2.24E-12	2.80E-09	9.77E-20	0.00E+00	0.00E+00	0.00E+00	0.00E+00
ZZQ-D3	27.11%	72.89%	0.00%	3.47E-09	5.42E-09	3.97E-10	1.79E-12	3.23E-23	3.54E-17	1.02E-10	3.70E-13	2.44E-09	7.21E-21	0.00E+00	0.00E+00	0.00E+00	0.00E+00
ZZQ-D4	24.68%	75.32%	0.00%	2.47E-09	4.77E-09	4.30E-10	1.27E-12	9.06E-24	1.56E-17	5.63E-11	2.21E-13	2.31E-09	3.44E-21	0.00E+00	0.00E+00	0.00E+00	0.00E+00
ZZQ-D5	32.69%	67.31%	0.00%	3.23E-09	5.18E-09	3.88E-10	1.17E-12	1.82E-23	2.63E-17	7.83E-11	2.78E-13	1.76E-09	5.24E-21	0.00E+00	0.00E+00	0.00E+00	0.00E+00
WGQ	95.13%	0.00%	4.87%	2.61E-09	2.50E-11	1.11E-14	0.00E+00	0.00E+00	2.90E-31	5.35E-27	5.41E-34	0.00E+00	5.41E-34	1.27E-10	8.45E-12	1.96E-13	1.81E-15
HMZP-L	100.00%	0.00%	0.00%	1.68E-07	2.92E-11	2.57E-16	0.00E+00	0.00E+00	2.99E-30	6.87E-28	1.62E-36	0.00E+00	1.62E-36	0.00E+00	0.00E+00	0.00E+00	0.00E+00
HMZP-LD1	100.00%	0.00%	0.00%	1.87E-07	1.51E-11	6.31E-17	0.00E+00	0.00E+00	7.65E-32	7.59E-30	4.39E-39	0.00E+00	4.39E-39	6.29E-12	2.92E-16	4.71E-21	3.02E-26
HMZP-LD2	100.00%	0.00%	0.00%	1.99E-07	1.49E-11	5.71E-17	0.00E+00	0.00E+00	5.90E-32	5.69E-30	2.98E-39	0.00E+00	2.98E-39	0.00E+00	0.00E+00	0.00E+00	0.00E+00
HMZP-LD3	100.00%	0.00%	0.00%	1.85E-07	3.85E-12	4.07E-18	0.00E+00	0.00E+00	1.65E-35	4.48E-34	0.00E+00	0.00E+00	0.00E+00	0.00E+00	0.00E+00	0.00E+00	0.00E+00
HMZP-M	100.00%	0.00%	0.00%	2.21E-07	7.77E-12	1.42E-17	0.00E+00	0.00E+00	1.46E-33	6.24E-32	0.00E+00	0.00E+00	0.00E+00	1.98E-13	2.46E-19	1.06E-25	1.81E-32
DGG	100.00%	0.00%	0.00%	4.20E-07	1.17E-11	1.90E-17	0.00E+00	0.00E+00	1.11E-31	2.26E-30	3.95E-40	0.00E+00	3.95E-40	2.30E-13	1.74E-19	4.57E-26	4.78E-33
YJQ-R	100.00%	0.00%	0.00%	3.72E-07	6.08E-13	5.65E-20	0.00E+00	0.00E+00	1.42E-39	1.92E-39	0.00E+00	0.00E+00	0.00E+00	1.53E-12	8.64E-18	1.70E-23	1.32E-29

Table S7. The EDX analysis results of pyrite and goethite before and after adsorbing the tungstate and polytungstate. The test points were marked by the blue dots in Fig. S5.

element	goethite Atomic%	Goethite,monotungstate Atomic%	Goethite,polytungstate Atomic%	pyrite Atomic%	pyrite,monotungstate Atomic%	pyrite,polytungstate Atomic%
S	—	—	—	47.04	44.90	43.06
Fe	31.34	30.85	28.01	25.09	24.99	29.70
O	68.54	67.67	60.71	5.01	10.9	5.82
W	0	0.18	0.31	0	0.18	0.12
Others	0.12	1.31	10.97	21.97	19.03	21.3

Reference

1. Schwertmann, U.; Cornell, R.M. *Iron Oxides in the Laboratory: Preparation and Characterization*; Wiley-VCH: Weinheim, New York, 2000.
2. Guo, Q.; Li, Y.; Luo, L. Tungsten from typical magmatic hydrothermal systems in China and its environmental transport. *Science of the Total Environment* **2019**, *657*, 1523-1534.
3. Smith, R.M.; Martell, A.E. NIST Critically Selected Stability Constants of Metal Complexes Database. **2004**.
4. Mohajerin, T.J.; Helz, G.R.; White, C.D.; Johannesson, K.H. Tungsten speciation in sulfidic waters: Determination of thiotungstate formation constants and modeling their distribution in natural waters. *Geochimica et Cosmochimica Acta* **2014**, *144*, 157-172.
5. Cruywagen, J.J.; van der Merwe, I.F. Tungsten (VI) equilibria: a potentiometric and calorimetric investigation. *Journal of the Chemical Society, Dalton Transactions* **1987**, *7*, 1701-1705.
6. Rozantsev, G.; Sazonova, O. Thermodynamic parameters of interconversions of isopolyanions in solutions of tungsten (VI). *Russian Journal of Coordination Chemistry* **2005**, *31*, 552-558.
